# GlnH, a Novel Antigen That Offers Partial Protection against Verocytotoxigenic *Escherichia coli* Infection

**DOI:** 10.3390/vaccines11010175

**Published:** 2023-01-13

**Authors:** Conor Quinn, Julen Tomás-Cortázar, Oritsejolomi Ofioritse, Joanne Cosgrave, Claire Purcell, Catherine McAloon, Susanna Frost, Siobhán McClean

**Affiliations:** 1 School of Biomolecular and Biomedical Sciences, University College Dublin, Belfield, Dublin 4, Ireland; 2 UCD Conway Institute, University College Dublin, Belfield, Dublin 24, Ireland; 3 APC Ltd., Building 11, Cherrywood Business Park, Loughlinstown, D18 DH5 Co. Dublin, Ireland; 4 Children’s Health Ireland (CHI) at Tallaght, Tallaght University Hospital, Tallaght, Dublin 24, Ireland; 5 School of Veterinary Medicine, University College Dublin, Belfield, Dublin 4, Ireland

**Keywords:** verocytotoxigenic *E. coli*, VTEC, vaccine, bacterial adhesin, adjuvants

## Abstract

Verotoxin-producing *Escherichia coli* (VTEC) causes zoonotic infections, with potentially devastating complications, and children under 5 years old are particularly susceptible. Antibiotic treatment is contraindicated, and due to the high proportion of infected children that suffer from severe and life-changing complications, there is an unmet need for a vaccine to prevent VTEC infections. Bacterial adhesins represent promising candidates for the successful development of a vaccine against VTEC. Using a proteomic approach to identify bacterial proteins interacting with human gastrointestinal epithelial Caco-2 and HT-29 cells, we identified eleven proteins by mass spectrometry. These included a glutamine-binding periplasmic protein, GlnH, a member of the ABC transporter family. The glnH gene was identified in 13 of the 15 bovine and all 5 human patient samples tested, suggesting that it is prevalent. We confirmed that GlnH is involved in the host cell attachment of an O157:H7 prototype *E. coli* strain to gastrointestinal cells in vitro. Recombinant GlnH was expressed and purified prior to the immunisation of mice. When alum was used as an adjuvant, GlnH was highly immunogenic, stimulating strong serological responses in immunised mice, and it resulted in a modest reduction in faecal shedding but did not reduce colonisation. GlnH immunisation with a T-cell-inducing adjuvant (SAS) also showed comparable antibody responses and an IgG1/IgG2a ratio suggestive of a mixed Th1/Th2 response but was partially protective, with a 1.5-log reduction in colonisation of the colon and caecum at 7 days relative to the adjuvant only (*p* = 0.0280). It is clear that future VTEC vaccine developments should consider the contribution of adjuvants in addition to antigens. Moreover, it is likely that a combined cellular and humoral response may prove more beneficial in providing protective interventions against VTEC.

## 1. Introduction

Verocytotoxin-producing *E. coli* (VTEC) are an important group of zoonotic pathogens that cause severe gastrointestinal infections with potentially life-threatening complications. Cattle act as asymptomatic carriers, allowing easy access into the environment that subsequently leads to infection in humans [1,2]. Ireland currently has the highest level of VTEC infection incidence in Europe, with an incidence rate of 17.5 cases per 100,000 population (2021, www.ecdc.europa.eu, accessed on 8 December 2022). The incidence and severity are highest in children under 5 years, as they are most likely to develop haemolytic uraemic syndrome (HUS), the leading cause of kidney failure among young children [3,4]. Antibiotic treatment is contraindicated for VTEC, and due to the high proportion of patients experiencing severe symptoms and VTEC-HUS complications, there is an unmet need for a vaccine to be produced against this infection [5,6,7,8,9,10,11,12,13]. 

VTEC can colonise the human intestinal tract via a range of surface colonisation factors, including components of type 1 to type 8 secretion systems, with different possible combinations modulating the virulence of some strains [14]. Adhesion and persistence are important features in bacterial pathogenesis, and it is widely recognised that reducing bacterial colonisation may protect susceptible populations from VTEC pathogenesis [15]. 

Vaccine development has focussed on verotoxin (vtx), which mediates cytotoxicity in the gut or kidney; and on the type 3 secretion system (T3SS) and associated proteins, with mixed results [16,17]. Initial efforts focussed on the ruminant reservoir. Recombinant subunit vaccines based on T3SS antigens, for example, have been effective in limiting *E. coli* colonisation and faecal shedding in cattle, e.g., Econiche, Bioniche Live Sciences, Ontario, Canada [18,19,20]. Adhesins also show promise as candidates for prophylactic vaccines against VTEC infections [21]; however, previously identified adhesins that are known to be involved in VTEC pathogenesis have not been found to be conserved across multiple VTEC serotypes [22], limiting their use as effective vaccine candidates. Intimin, for example, is a potent immunogenic adhesin [23], which elicits specific antibodies in patients who have developed haemorrhagic colitis and HUS [24,25] but has at least 27 variants comprising 18 types and 9 subtypes [26]. Antibody-mediated intimin protection is specific to only two intimin subtypes, β and γ [22], which may impede its use as a vaccine antigen [27]. DNA vaccine approaches have been developed that elicited Th2 cytokine responses and reduced caecal colonisation by 1 log [28]. Subsequent gold-nanoparticle formulations of the subunit protein antigens LomW or EscC resulted in a 1- to 1.5-log reduction in colon colonisation at day 3 [29]. A variety of fusion protein constructs of inactivated vtx proteins fused to adhesins and other antigens have stimulated strong serological responses and increased survival in mice, as recently reviewed [15], but none of these developments have been approved to date. Moreover, recent alternative platforms have been examined, including *Lactobacillus acidophilus* strains decorated with EspA, intimin, Tir and H7 Flagellin, and they showed a reduction in fecal shedding [30] at day 11 post-challenge. A salmonella-based oral vaccine bearing the same four antigens stimulated mucosal antibody responses and showed a reduction in bacterial shedding at day 6 [31]. Outer membrane vesicle (OMV) formulations of VTEC strains protected against pathogenicity in a murine model and against a lethal shigella toxin challenge [32]; however, there may be considerable safety concerns with this approach. 

There is scope to discover and develop new or improved vaccine antigens. VTEC harbours multiple proteins with varying degrees of interaction with the intestinal epithelia; thus, it is highly probable that novel protein adhesins have yet to be discovered, which may be common among O157 and non-O157 pathogenic *E. coli* strains, and which may improve the breadth or depth of protection of existing vaccine candidates [26,33]. We have previously developed a proteomic cell blot approach to identify bacterial proteins involved in epithelial cell attachment with a high potential to be effective vaccine antigens [34]. Using this approach, we have already identified an effective melioidosis vaccine candidate that protected mice for up to 81 days against *Burkholderia pseudomallei,* and that will be progressing to human studies in the near future [35,36]. Our aim was to extend this platform to identify novel protein adhesins specific to VTEC strains that may be effective vaccine antigens, either on their own or in combination with other antigens, to prevent human colonisation. The focus on human colonisation is warranted, given that the vaccination of cattle to prevent their colonisation has minimal economic benefit to meat producers. We have identified several VTEC proteins that had an ability to attach to human gastrointestinal cells, and we evaluated the protective efficacy of one of these in challenge studies. 

## 2. Materials and Methods

### 2.1. Bacterial Strains

The non-VTEC-producing *E. coli* O157:H7 prototype strain NCTC 12900 and commensal *E. coli* strain HS that were used in this study were routinely cultured in Luria Bertani (LB) broth at 37 °C with agitation at 200 rpm. The NCTC 12900 Nal^r^ strain that does not possess vtx1 or vtx2 genes was chosen for biosafety reasons. Its colonisation of ruminants and its interactions with bovine epithelial cells have been reported [37,38].

### 2.2. Human Cell Lines and Maintenance

Two human gastrointestinal cell lines were used, human colorectal adenocarcinoma cells, and HT29, and human colon carcinoma cells, Caco-2. HT-29 cells were maintained in McCoy’s 5a media supplemented with 10% foetal bovine serum (FBS) (Sigma Aldrich, St. Louis, MO, USA), 1% penicillin/streptomycin and 1% L-glutamine. The Caco-2 cells were maintained in Dulbecco’s modified Eagle’s medium (DMEM) supplemented with 10% FBS, 1% penicillin/streptomycin, 1% L-glutamine and 1% non-essential amino acids. Prior to use, the cells were washed with sterile PBS, before being detached from the culture flasks with 0.05 % porcine trypsin (Sigma Aldrich, St. Louis, MO, USA) in phosphate-buffered saline PBS for 5 min at 37 °C in 5% CO2. The trypsin was deactivated by the addition of fresh prewarmed media, and the disaggregated cells were harvested by centrifugation at 700× *g* for 5 min and resuspended in fresh medium at the required dilution. 

### 2.3. Attachment of E. coli Strains to Gastrointestinal Epithelial Cells

Host cell attachment was determined according to previously published methods [34]. Human Caco-2 or HT-29 epithelial cells were seeded at a concentration of 4 × 10^5^ cells/well overnight in 24-well plates in fresh media. Bacteria were grown to the mid-logarithmic phase and diluted to 2 × 10^7^ bacterial cells/well in 1 mL of DMEM. Caco-2 or HT-29 cells were washed with 1 ml of warm media before the addition of bacteria in a final volume of 1ml in duplicate. The 24-well plates were centrifuged at 700× *g* for 5 min to promote adhesion and incubated for 30 min at 37 °C. The wells were washed four times with warm sterile phosphate-buffered saline (PBS) before the incubation with 500 μL lysis buffer (125 μL Triton X-100 in 50 mL PBS) for 20 min at room temperature. The cells were removed by scraping with a pipette tip, serially diluted up to a factor of 10^−4^, plated onto LB agar and incubated overnight at 37 °C, after which colonies were counted. 

### 2.4. Outer Membrane Protein (OMP) Preparation and Quantification 

Membrane proteins were prepared for 2D electophoresis using an adapted method from Montero et al. [39]. Overnight cultures of bacterial strains were pelleted at 4 °C and resuspended in lysis buffer containing 0.01 M 4-(2-hydroxyethyl)-1-piperazineethanesulfonic acid (HEPES) solution, 800 µL EDTA-free protease inhibitor cocktail and 10 mg/mL lysozyme and incubated at 37 °C for 2 h at 80 rpm. After incubation, 4% triton X-100 was added and the cells exposed to cycles of bead beating on ice for 2 min, followed by 3 min at rest, at 5 min intervals for one hour. The supernatants were recovered by centrifugation at 4 °C, placed in fresh tubes and centrifuged to remove cell debris at 12,000× *g* for 10 min at 4 °C. The pellets were discarded, and supernatant proteins were solubilised in 15 ml 2% Sarkosyl (N-lauroylsarcosine sodium salt) at 25 °C for 30 min, followed by ultracentrifugation at 20,500× *g* for 80 min at 4 °C. The OMP-enriched pellets were resuspended in 1 ml filtered deionised water. Aliquots of 100 µL of membrane protein preparation were removed to estimate the protein concentration using a bicinchoninic acid (BCA) (Thermo Fisher Scientific, Carrigaline, Co Cork, Ireland). A second ultracentrifugation step was carried out on the remaining 900 µL at 20,500× *g* for 50 min at 4 °C, and pellets were resuspended in 1 mL 2D lysis buffer containing 8 M urea, 2 M thiourea, 4% 3-((3-cholamidopropyl)dimethylammonio)-1-propanesulfonic acid (CHAPS), 1% triton X-100, 10 mM Tris Base, 65 mM dithiothreitol (DTT) and 0.8% immobilised pH gradient (IPG) buffer in ultra-pure water. 

### 2.5. Protein Separation by 2-D Gel Electrophoresis

OMP-enriched preparations for each strain were solubilised for isoelectric focusing (IEF) in rehydration buffer to a total volume of 125 µL, containing 8 M urea, 2 M thiourea, 4% CHAPS, 1% Triton X-100, 10 mM Tris base, 65 mM DTT and 0.8% (*v*/*v*) IPG buffer (pH 3–11 NL) with a trace of bromophenol blue. IPG strips (pH 3–11) NL of 7 cm were rehydrated overnight with 125 µL of rehydration solution containing 200 µg of proteins. IEF was performed for a total of 3 h using the following conditions: 500 V for 30 min, 1000 V for 30 min and 5000 V for 2 h. Following IEF, IPG strips were incubated at room temperature for 15 min in equilibration buffer containing 2% DTT, 30% glycerol, 2% SDS, 6 M urea and 50 mM Tris, and the proteins were alkylated for an additional 15 min in the same buffer containing 2.5% iodoacetamide instead of DTT. IPG strips were placed on 12% SDS-PAGE gels, sealed with agarose, and separation was performed for 20 min at 80 V, followed by an additional 90 min at 120 V. The polyacrylamide gels were then equilibrated in transfer buffer for 15 min prior to transfer to polyvinylidene difluoride (PVDF) membranes on a semi-dry transfer cell (Bio-Rad, Watford, UK) at 20 V for 2 h.

### 2.6. Gastrointestinal Epithelial Cell-Probed Blots 

Proteins involved in intestinal cell attachment were detected using a modification of our earlier cell blot proteomic approach [34]. The 2D membranes were blocked overnight at 4 °C with 5% BSA and 3% Marvel in PBS with rotation (60 rpm). Human intestinal epithelial cells, either Caco-2 or HT-29, were scraped from T75 culture flasks, resuspended at 1 × 10 ^6^ cells/mL in 10 mL PBS and incubated with the PVDF membranes for 4 h at 37 °C with gentle agitation (60 rpm). The membranes were then rinsed with PBS, and the epithelial cells were fixed with 3% paraformaldehyde in PBS for 10 min. Bound cells were detected with an anti-epithelial-specific antigen antibody (VU-1D9 clone (Merck), 1:1000 in 5% (*w*/*v*) BSA, 0.5% PBS-T) incubated overnight at 4 °C with end-to-end rotation. The membranes were then washed three times with 0.5% (*v*/*v*) PBS-T before being incubated with a secondary HRP-conjugated anti-mouse antibody (1:36,000 dilution in 5% BSA, 0.5% PBS-T) for 1 h at room temperature with end-to-end rotation. The membranes were subsequently washed 5 times with 0.5% PBS-T, before chemiluminescence detection with luminol reagent, then incubated for 5 min at room temperature, followed by exposure to film (20–30 min). Spots on developed membranes were matched with spots identified on a Page Blue^TM^ (Thermo-Fisher, Waltham, MA, USA)-stained gels that had been run in parallel [34], and they were excised for MS analysis. 

### 2.7. Tryptic Digestion of Protein Samples and Preparation for LC/MS 

Tryptic digestion of proteins was performed following the in-gel digestion method described by Shevchenko et al. [40]. The proteins were redissolved in 20 µL of 0.1% (*v*/*v*) trifluoroacetic acid (TFA) prior to Zip-tip clean up and identified in technical triplicates with a Thermo Scientific Q Exactive mass spectrometer connected to a Dionex Ultimate 3000 RSLC nano chromatography system. Proteins were separated on a C18 column (C18RP Reposil-Pur, 100 × 0.075 mm × 3 μm) over 60 min at a flow rate of 250 nL/min with a linear gradient of increasing acetonitrile from 1% to 27%. The mass spectrometer was operated in data-dependent mode; a high-resolution (70,000) MS scan (300–1600 *m*/*z*) was performed to select the twelve most intense ions and fragmented using high-energy C-trap dissociation for MS/MS analysis. Raw data from the Q-Exactive was processed using MaxQuant [41,42] (version 1.6.3.4), incorporating the Andromeda search engine [43]. MS/MS spectra were matched against the *E. coli* O157:H7 NCTC12900 database. The sequence coverage threshold was set at ≥40%, and the number of unique peptides threshold was set at >5 for selection. 

### 2.8. PCR Analysis of Bovine and Human VTEC Isolates

Bovine faecal samples were obtained from the University College Dublin, School of Veterinary Medicine (UCD SVM), plated and cultured overnight at 37 °C on Sorbitol MacConkey (SMAC) agar. Five VTEC-positive cultures from VTEC-positive patients identified at Tallaght University Hospital were also plated on SMAC overnight. DNA templates from presumptive VTEC isolates were prepared by boiling individual colonies at 95 °C for 10 min. PCR reaction master mix containing HotStar Taq Master Mix taq polymerase (25 µL), sterile water (15 µL) and forward (CACCATGAAGTCTGTATTAAAAGT) and reverse (TTATTTCGGTTCAGTACCGA) primers (2.5 µL each) was mixed with either DNA template (5 µL) or water as a control (5 µL). Reactions were run with the following conditions: 95 °C denaturation, 58 °C annealing and 72 °C extension for a total of 38 cycles. Following PCR, samples were mixed with Thermo Scientific^TM^ TriTrack DNA loading dye and run on 2% agarose gel in 1X TBS at 100 V for 1 h. 

### 2.9. Cloning of glnH Gene

Primers for *glnH* were designed using a BLAST NCIB search of Uniprot ID P0AEQ5 gene name GlnH/ECs0889 against *Escherichia coli* O157:H7 Sakai genome sequence NC_002695.1 (Table 1). The forward primer (CACCATGAAGTCTGTATTAAAAGT) included the four-base sequence ‘CACC’ to allow for directional cloning of the *glnH* gene into the pET100/D-TOPO vector. The reverse primer (TTATTTCGGTTCAGTACCGA) was designed to amplify the gene of interest with a product of 747 bp, and the vector incorporated an N-terminal 6x-his tag. The gene was amplified by PCR and verified on 2% agarose gels, before the *glnH* construct was cloned into OneShot TOP10 chemically competent cells, as per the manufacturer’s instructions (ThermoFisher, Waltham, MA, USA), plated on LB agar supplemented with 50 µg/mL carbenicillin (LB Carbo_50_) and incubated overnight. Confirmation of successful insertion was confirmed by PCR and directional sequencing. PCR-positive colonies were used to transform the *E. coli* BL21 Star DE3 strain. Overnight cultures were pelleted, and the pET100/D-TOPO vector containing *glnH* was extracted using a QIAprep Spin miniprep kit (Qiagen). The eluted plasmids (5 µL) were transformed into BL21-competent *E. coli* cells by heat-shock treatment. The cells were then plated and incubated overnight on LB Carbo_50_ plates, and the successful transformation of colonies was confirmed by colony PCR. 

### 2.10. Host Cell Attachment of E. coli BL21 Strain Expressing Recombinant GlnH 

Gastrointestinal cells (HT29 or Caco-2) were seeded overnight in 24-well plates at a density of 4 × 10^5^ cells/well. Overnight cultures of *E. coli* BL21 transformed with the *glnH* gene were re-inoculated into 100 mL LB broth containing 50 µg/mL carbenicillin and cultured for 2 h at 37 °C, and GlnH expression was induced by the addition of IPTG (1 mM) for 3 h. Epithelial cells were washed with fresh media (McCoy’s or Dulbecco’s minimal essential medium (DMEM) for HT29 and Caco-2, respectively), and the induced and uninduced cultures were resuspended in the relevant media added to each well at an MOI of 25:1 (1 × 10^7^ CFU/well) and the plates centrifuged at 700× *g* for 5 min, before being incubated at 37 °C and 5% CO_2_ for 30 min. The wells were washed four times in sterile PBS to remove unattached bacteria, and the remaining bacteria were detached with 0.25% Triton X-100 for 20 min at room temperature, as described previously [34]. Wells were scraped using fresh pipette tips, and the contents of each well were serially diluted and plated onto LB agar supplemented with 50 µg/mL carbenicillin. Plates were incubated overnight at 37 °C, and the number of bacterial cells adhering was determined by plate counting. 

### 2.11. GlnH Expression and Purification 

Overnight cultures (100 mL) containing *E. coli* BL21 cells transformed with pET-D/Topo vectors containing the *glnH* gene were re-inoculated into 2 litres LB broth supplemented with 50 µµg/mL carbenicillin and cultured for two hours to the mid-log phase at 37 °C with shaking at 200 rpm. Cultures were induced with 0.1 mM IPTG for 5 h at 37 °C at 200 rpm. Bacterial cells were pelleted at 2500× *g* for 10 min at 4 °C, and the cell pellets were resuspended in 100 mL Ni-NTA lysis buffer containing 50 mM Tris HCl, 300 mM NaCl, 10% glycerol and 10 mM Imidazole, pH 8.8 with 5N NaOH, with protease inhibitor cocktail and lysozyme (25 µg/mL). The lysate was incubated on ice with mild rotation using a magnetic stirrer for 15 min before being sonicated with ten fifteen-second pulses and one-minute rest intervals on ice. After sonication, protein samples were ultracentrifuged at 10,000× *g* for 1 h at 4 °C. Ni-NTA resin (Thermo Fisher, Waltham, MA, USA) was equilibrated by rinsing with milli-Q water followed by Ni-NTA lysis buffer, before supernatant was incubated with the resin overnight at 4 °C with mild rotation using a magnetic stirrer. After overnight incubation, the resin was passed through a column, before being washed with 250 mL wash buffer containing 50 mM Tris HCl, 300 mM NaCl, 10% glycerol and 10 mM imidazole, with a pH 8.8. The GlnH protein was eluted stepwise with 50 mM Tris HCl and 300 mM NaCl containing 250 mM Imidazole or 300 mM Imidazole, with 10% glycerol and a pH 8.8. The eluted fractions were concentrated using Amicon Ultra-cell 3-10K filters before further purification by size exclusion chromatography (SEC) on a pre-equilibrated Hi load 16/60 Superdex 75 SEC column on an AKTA Explorer. Fractions of 1.8 mL were collected, and absorbance was read at 280 nm. Aliquots of each fraction were collected for visualisation on SDS-PAGE, and each fraction was pooled together for further analysis. 

### 2.12. Preparation of Nal^r^-Resistant Prototype Strain E. coli O157 Strain NCTC 12900

The Shiga toxin-negative *E. coli* O157 prototype strain NCTC 12900 was made resistant to Nalidixic acid sodium salt (Sigma Aldrich, St. Louis, MO, USA, product code N4382-5G) by passage on SMAC agar supplemented with 15 µg/mL nalidixic acid and a designated Vtx-negative *E. coli* O157 prototype strain NCTC12900 Nal^r^ [37]. NCTC12900 Nal^r^ bacterial stocks were prepared by the inoculation of colonies positive for Nal^r^ resistance into 10 mls of LB broth supplemented with 15 µg/ml Nalidixic acid sodium salt and incubated overnight at 37 °C at 200 rpm. Five ml aliquots were re-inoculated into 100 mL LB broth supplemented with 15 µg/mL nalidixic acid sodium salt and grown to an optical density (OD) of 0.4–0.8. Cells were pelleted by centrifugation at 2500× *g* for 10 min, before being resuspended in LB broth containing 20% glycerol. Stocks were stored at −80 °C until needed. 

### 2.13. Immunisation with GlnH and Bacterial Challenge in BALB/c Mice

Groups of 6- to 8-week-old BALB/c mice (5 mice per group, from Charles River, UK) were used to evaluate the protective efficacy of GlnH. Groups of BALB/c mice were immunised with either 10 µg or 50 µg of GlnH mixed 1:1 with alum; or mixed 1:1 with Sigma adjuvanted system (SAS); alum alone; SAS alone; or PBS alone in a total volume of 100 µL on day 0, followed by two booster immunisations on days 14 and 28. Subcutaneous administration involved two 50 µL doses on both sides of the mouse abdomen to maximise uptake and immune response. Fourteen days after the final immunisation, food was restricted for 12 h prior to bacterial challenge. The mouse challenge model was first established by administering a range of CFU (4 × 10^4^ to 4 × 10^9^ CFU) of NCTC 12900Nal^r^ by oral gavage to cimetidine-treated mice and monitoring faecal shedding for up to 7 days and colon colonisation at 7 days. Mice were administered 50 mg/kg cimetidine orally to reduce stomach acidity 2 h before being challenged with 400 µL of NCTC 12900Nal^r^ (4 × 10^9^ CFU) in PBS via oral gavage. Mice were monitored for 7 consecutive days until euthanasia by carbon dioxide exposure, after which the caecum and colon were dissected. The level of O157 colonisation was enumerated by homogenising tissue samples of known length and weight in a TissueLyser (Qiagen) at 30 Hz for 20 min, removing debris by centrifugation at 600× *g* for 3 min, followed by serial dilution and plating on MacConkey agar with Sorbitol (SMAC) supplemented with 15 g/mL nalidixic acid. Bacteria were incubated overnight at 37 °C and counted the following day. To determine faecal shedding, two 0.1 g samples of faecal pellets were collected daily from each treatment groups’ cage post-bacterial challenge and homogenised by vigorous vortexing in 1 mL PBS, before being left to stand to separate debris by sedimentation. Faecal homogenates were serially diluted in Ringer’s solution to 10^−6^ and plated (50 uL) on MacConkey agar with Sorbitol supplemented with 15 µg/mL Nalidixic acid. Plates were counted on the following day, representative of the number of bacteria shed daily per gram of faeces from O157-infected mice in each cage. 

### 2.14. Serum IgG Antibodies Determination

Blood was collected from each mouse via cheek bleeds one week after the second booster for serological analysis of the immune response against GlnH immunisation. The blood was allowed to stand at 4 °C overnight before being centrifuged for 30 min to isolate serum, which was transferred to freshly labelled tubes and stored at −80 until required. Microtitre plates were coated overnight with 100 µL of purified GlnH antigen (0.5 µg/mL) in 0.2 M sodium carbonate pH 9.6 at 4 °C. Plates were washed 3 times with PBS containing 0.05% Tween-20 (PBS-T) and blocked with PBS containing 1% FBS for 1 h at room temperature. Four-fold dilutions of sera were added to wells in triplicate and incubated for two hours at room temperature. The wells were washed 3 times in PBS-T and incubated in 100 µL of HRP-conjugated anti-mouse antibodies specific for total IgG, IgG1 or IgG2a (diluted 1:5000 in PBS containing 1% FBS) at room temperature for 60 min. Plates were washed again as before, followed by two additional washes containing 400 µL/well of 1 X PBS. TMB substrate (100 µL) was added to each well, and the plates were incubated at room temperature until a colour change was observed, at which time reactions ceased by using 100 µL/well of 2 M sulfuric acid. Plates were read at an absorbance of 450 nm within 30 min. Serum antibody titres were defined as endpoint titres, i.e., the reciprocal of the highest dilution of serum producing an OD above the cutoff value, where the cutoff values were determined as the OD of the corresponding dilution of control sera plus 3 standard deviations. 

### 2.15. Statistical Analysis

Statistical analysis of host cell attachment and colonisation of the mouse caecum and colon were performed by one-way ANOVA with Sidak’s multiple comparison test. Statistical analysis of faecal shedding was performed using two-way ANOVA Tukey’s multiple comparison test. Analysis of weight gain over 7 days was performed by Two-way ANOVA with Sidak’s multiple comparison test. All statistical analysis and area under the faecal curve calculations were performed using GraphPad Prism (software version 9). 

## 3. Results

### 3.1. Comparison of Attachment of Commensal and VTEC Prototype Strains to Gastrointestinal Epithelial Cells

**Figure 1:**
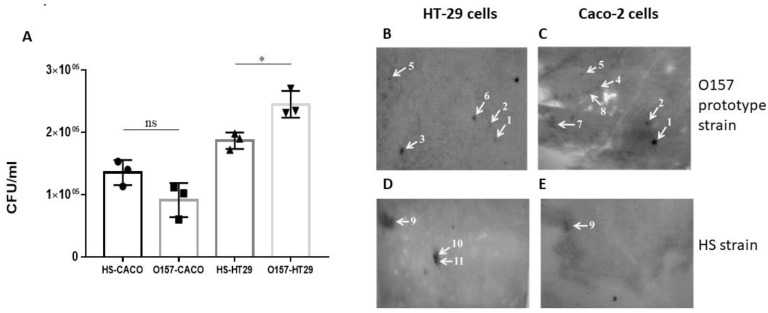
Identification of proteins involved in VTEC and commensal *E. coli* attachment to gastrointestinal cells by a cell blot approach. (**A**) Comparison of attachment of NCTC12900 and HS *E. coli* strains to gastrointestinal epithelial cells. Data represent mean ± SEM of three independent experiments of each bacterial strain and gastrointestinal cell line. * Statistical analysis performed using one-way ANOVA with Sidak’s multiple comparison test. (ns: indicates not statistically significant); (**B**–**E**) Representative cell-probed blots prepared from O157:H7 NCTC 12900 (**B**,**C**) and the commensal *E. coli* HS (**D**,**E**) cells. Blots were probed with 1 × 10^6^ cells/mL HT29 (**B**,**D**) or Caco-2 (**C**,**E**) cells, respectively. Identified proteins are highlighted with white arrows, and numbers correspond to those listed in Table 1.

The independent human gastrointestinal epithelial cell lines HT29 and Caco-2 were used to examine the attachment of the NCTC 12900 O157 prototype strain and a commensal HS *E. coli* strain to host cells. The O157 strain showed a level of greater attachment to HT29 cells compared to the commensal strain (* *p* = 0.0193). In contrast, the level of attachment of the *E. coli* HS strain and the O157 prototype strain to Caco-2 cells was comparable (Figure 1A).

### 3.2. Identification of Proteins Involved in Gastrointestinal Cell Attachment

To identify proteins involved in the attachment of *E. coli* bacteria to host cells, membrane protein-enriched fractions prepared from the O157:H7 *E. coli* strain, NCTC12900 or the commensal *E. coli* HS strain that were separated and transferred onto PVDF membranes were probed with either HT29 or Caco-2 intestinal epithelial cells. Several spots that were highlighted on the cell blots shared common locations with blots prepared from the two independent epithelial cell lines, and some were unique to a specific cell line (Figure 1). In total, eleven distinct proteins were reproducibly identified, four of which were common to both HT29 and Caco-2 blots (Table 1). There were six proteins reproducibly identified on Caco-2-probed blots prepared from the VTEC strain O157:H7 (Figure 1B), three of which were also identified in HT29-probed blots (Figure 1C). Eight proteins identified in *E. coli* O157 attachment to host cells (HT29 and Caco-2) were within the molecular mass range of 20 to 64 kDa and included proteins with known immunogenicity and roles in host attachment or pathogenesis of other bacterial species, including alkyl hydroperoxide reductase C (AhpC) and Enolase [44,45,46,47]. Other proteins identified had not been shown previously to be involved in host attachment of VTEC, including the Glutamine-binding periplasmic protein (GlnH) (Table 1; Figure 1B,C). This protein and two others, FKBP-type peptidyl-prolyl cis-trans isomerase FkpA and Phosphoenolpyruvate-protein phosphotransferase (PPP), were identified in both Caco-2 and HT29-probed blots, and the remaining membrane proteins identified were exclusive to either HT29 or Caco-2 blots (Table 1). There were also three proteins identified from HT29-probed blots prepared from membrane proteins of the commensal *E. coli* strain HS, all in the molecular mass range of 39 to 60 kDa (Figure 1E), which were also identified in Caco-2 blots: outer membrane protein C (OmpC), outer membrane protein A (OmpA) and flagellin (Table 1; Figure 1). Based on VTEC O157:H7 exclusivity (i.e., the absence of detection on HS strain blots); HT29 and Caco-2 cell interaction; percentage of sequence coverage (77.8%); the number of unique peptides (21); and lack of information known about its role in host cell attachment, the GlnH protein was selected for further analysis (Table 1). 

### 3.3. The GlnH Protein Plays a Role in E. coli Attachment 

**Figure 2:**
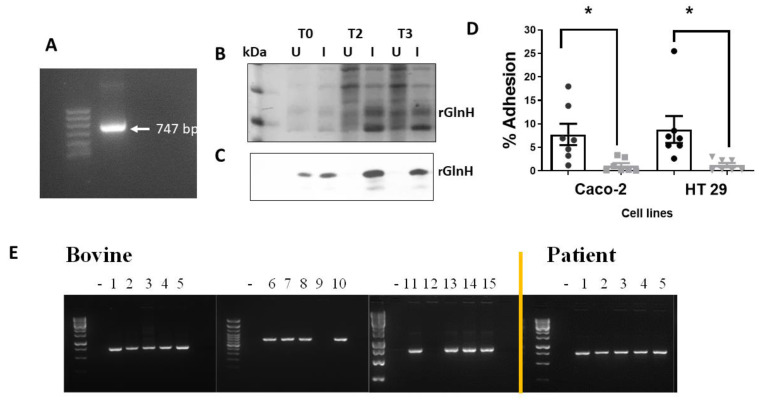
Effect of expression of GlnH on host cell attachment and prevalence of *glnH* gene in bovine and human patient isolates. (**A**) The *glnH* gene was cloned into BL_21 cells and its presence confirmed by PCR analysis, identifying an amplicon at 747 bp. Expression of GlnH in BL21 cells after 3 h induction with IPTG (1 mM) was confirmed by SDS-PAGE gel stained with Page Blue^TM^ (**B**) and Western blot using an anti-His-tag antibody (**C**). (**D**) Expression of GlnH protein increased the level of bacterial attachment to gastrointestinal cells compared to uninduced BL21 cells in vitro. The level of attachment of induced (black) and uninduced (grey) BL21 cells recombinantly expressing protein GlnH to gastrointestinal cells (HT29 and Caco-2). Each error bar represents mean ± SEM from four independent experiments. Black bars represent induced BL21_*glnH* cells, and grey bars represent BL21_*glnH* cells that were not induced. Differences in % adhesion were analysed by one-way ANOVA with Sidak’s multiple comparison test (*, *p* < 0.05). (**E**) Presence of the *glnH* gene identified in VTEC isolates from 15 bovine and 5 human patient isolates, as determined by PCR amplification and visualisation on 2% agarose gels.

Prior to immunisation with purified rGlnH, we wished to confirm that the protein was involved in host cell attachment. The *glnH* gene was amplified and cloned into a pET100 vector to enable expression in BL21 cells, and successful transformation was confirmed by an amplicon at 747 bp (Figure 2A). Recombinant GlnH expression was induced in BL21_*glnH* cells for 3 h with IPTG and expression was confirmed by Western blot (Figure 2B,C). Induced BL21_*glnH* cells recombinantly expressing the rGlnH showed a four-fold increase in the level of bacterial attachment to both Caco-2 and HT29 epithelial cells compared to uninduced BL21 cells (*p* = 0.0371; *p* = 0.0161, respectively, Figure 2D). This confirms that GlnH is involved in host cell attachment. 

### 3.4. Presence in Bovine and Human Patient Samples

The presence of the *glnH* gene in a limited random selection of bovine and human patient faecal samples was assessed prior to evaluation for potential efficacy to ensure that the gene was present in colonised cattle and in patient samples. Fifteen bovine presumptive VTEC isolates based on growth on SMAC agar overnight at 37 °C and five samples from VTEC-positive patients identified at the Tallaght University Hospital were screened for the presence of the *glnH* gene. The *glnH* gene was identified in 13 out of 15 bovine samples and in all 5 human VTEC patient isolates examined (Figure 2E). 

### 3.5. Purification of the Recombinant GlnH Protein for Preclinical Evaluation

**Figure 3:**
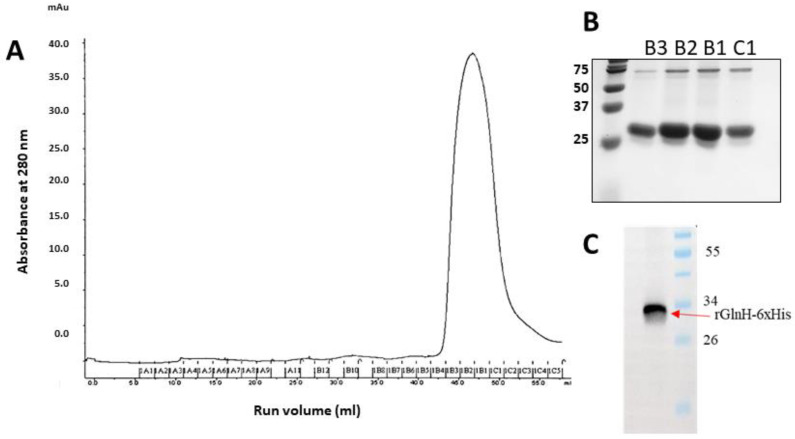
Purification of rGlnH continued by size exclusion (SE) chromatography. The secondary purification of expressed and affinity-purified rGlnH using a HiLoad^®^ 16/600 Superdex^®^ 75 pg column 16/60. Fractions collected in 96-well plate representative of large peak at a run volume (mL) 43–55 mL (**A**) and the purity of fractions B3, B2, B1 and C1 was visualised on 12% SDS gel (**B**). (**C**) Western blot confirms presence of His-tagged rGlnH.

Optimal recombinant GlnH expression was observed five hours after induction with 0.1 mM IPTG. Following purification on a NiNTA column, the fractions containing eluted rGlnH were pooled, concentrated and further purified by SEC, as indicated by a single large peak on chromatograms between fractions B3 and C2 represented the GlnH protein (Figure 3A), and its purity was determined by SDS-PAGE (Figure 3B). Aliquots of rGlnH from the SE chromatography eluants ranged in concentration from 600 µg/mL to 930 µg/mL. The endotoxin level in purified GlnH was 42.7 EU/mL, equivalent to 0.41 EU/10 µg dose and consequently below the recommended limit for mouse immunisation [48]. The purified (His)-tagged rGlnH protein was visualised by Western blotting (Figure 3C), and the identification of purified antigen was further confirmed by MS analysis (29 peptides matched; sequence coverage 81%). 

### 3.6. GlnH Is Immunogenic in Immunised Mice

**Figure 4:**
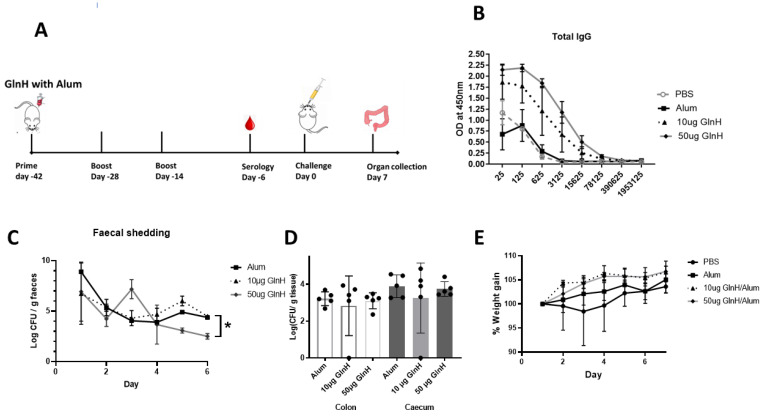
(**A**) Timeline of vaccination schedule, indicating days of immunisation, challenge and organ collection. Mice were immunised three times with 10 or 50 µg recombinant GlnH antigen with alum as adjuvant. (**B**) Serological analysis of responses to GlnH immunisation in mice. Sera were obtained by blood collection seven days after third immunisation dose, serially diluted and antibody titres determined by ELISA using antigen-specific antibodies and antibodies specific for mouse IgGs. Data are reported as the OD obtained at each dilution. (**C**) Faecal shedding was monitored daily to access protection conferred against *E. coli* O157 NCTC Nal^r^ 6 days post-challenge. The daily *E. coli* O157 shed from mice faeces (pooled per cage) are represented as mean CFU ± SD of two independent faecal collections from each group. Significant differences in daily NCTC12900Nal^r^ shed between groups were determined via two-way ANOVA Tukey’s multiple comparison test (*, *p* < 0.05). (**D**) Colonisation of colon (white bars) and caecum (grey bars) at day 7 post-challenge with 4 × 10^9^ CFU with *E. coli* prototype strain NCTC 12900 Nal^r^; the colon and caecum were collected from each mouse after euthanasia. Bacterial burden was determined per gram of tissue, and level of colonisation was expressed in log scale. All colonisation data are displayed as means ± SEM of results obtained per treatment group. No statistical significance was observed by analysis using a one-way ANOVA with Sidak’s multiple comparison test between immunised and non-immunized groups. (**E**) Weight gain in immunised mice relative to control mice over 7 days post challenge. Data are normalised relative to individual mouse weight.

To examine the impact on mouse colonisation, we developed a mouse challenge model with the NCTC12900Nal^r^ strain (Supplemental Figure S1A). We observed that mice challenged with 4 × 10^9^ CFU shed NCTC12900Nal^r^ cells over a period of 7 days (Supplemental Figure S1B). Moreover, 80% of mice maintained NCTC12900Nal^r^ colonisation at day 7 (Supplemental Figure S1C). Mice were immunised three times before oral challenge, as indicated in Figure 4A. Serological analysis showed that the GlnH antigen was quite immunogenic when used to immunise mice with alum as an adjuvant. Total IgG antibody titres of log_10_ of 6.273 were achieved (Figure 4B). 

**Figure 5:**
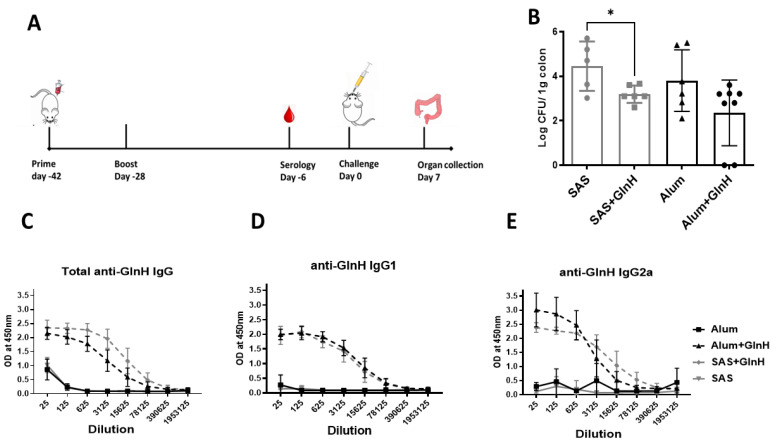
(**A**) Timeline of vaccination schedule, indicating days of immunisation, challenge and organ collection. Mice were immunised twice with 10 µg recombinant GlnH antigen with alum or SAS as adjuvant. (**B**) Protection of mice from colonisation following immunisation with recombinant GlnH with SAS as adjuvant. Mice were challenged by oral gavage with 4 × 10^9^ CFU *E. coli* prototype strain NCTC 12900 Nal^r^, and the colons including caeca were collected from each mouse after euthanasia. Bacterial burden was determined per gram of tissue, and level of colonisation was plotted in log scale. All colonisation data are displayed as means ± standard errors of the mean (SEM) of results obtained per treatment group. * Significant difference of NCTC 12900 Nal^r^ colonisation in the GI tract of mice (*p* = 0.028). (**C**–**E**): GlnH-specific total IgG (**C**), IgG1 (**D**) and IgG2a (**E**) responses were measured in mice immunised twice with 10 µg GlnH in the presence of either SAS or alum and compared with respective adjuvant-only groups by indirect ELISA. Sera were obtained by blood collection 28 days after second immunisation dose.

GlnH-/Alum-immunised mice challenged with the NCTC12900Nal^r^ O157 prototype strain showed modest protection from *E. coli* O157:H7, as shown by reduced faecal shedding on day 6 p.i. (Figure 4C, *p* < 0.0218) and reduced area under the curve overall (29.6 versus 25.6 CFU days/g tissue, for Alum- and GlnH-immunised, respectively. However, GlnH/Alum failed to protect mice from colonisation (Figure 4D). Despite a lack of change in faecal shedding, the immunised mice gained more weight post-challenge relative to the alum control group (Figure 4E, *p* = 0.0296), suggestive of better wellbeing. Cellular immune responses are involved in protection against VTEC; consequently, we examined whether an adjuvant that stimulated T-cell activation would enhance *E. coli* O157Nal^r^ protection over robust antibody production. We chose the T-cell-stimulating adjuvant, SAS, which comprises monophosphoryl lipid A and trehalose dimycolate [35]. Mice immunised s.c. with 10 µg GlnH in combination with either SAS (1:1) or alum (1:1) were compared to mice treated with either adjuvant alone. The GlnH protein was again confirmed as a highly immunogenic B-cell antigen, with strong antibody titres following immunisation with 10 µg rGlnH regardless of the adjuvant (Log_10_ 6.97, Figure 5A), indicating that the adjuvant did not dramatically affect serological responses. IgG1 responses were marginally higher in GlnH-/Alum-immunised mice relative to GlnH-/SAS-immunised mice (log_10_ 6.972 versus log_10_ 6.273), whereas GlnH-specific IgG2a titres were higher in the GlnH-/SAS-immunised group (log_10_ 6.97) relative to the GlnH adjuvanted with the alum group (log_10_ 6.27), suggesting slight skewing of a Th 1 helper response when SAS was used as the adjuvant. A 1.5-fold reduction in gastrointestinal colonisation (caecum and colon tissues combined, *p* = 0.0280) was observed at 7 d.p.i in GlnH-/SAS-immunised mice compared to the SAS-alone group (Figure 5D), and there was no statistically significant reduction in colonisation in the GlnH-/Alum-immunised mice relative to the alum-only control group. 

## 4. Discussion

To date, vaccine development against VTEC has been limited to a relatively small number of virulence factors that tend to be subtype specific and predominately focus on the production of high levels of antibodies (Th2 humoral response) to confer protection [15,16]. Bacterial proteins involved in host cell attachment make good vaccine candidates; thus, we aimed to identify previously unidentified proteins in VTEC that are involved in that process. Using a 2D cell blot proteomic approach [34], GlnH was one of several antigens identified as being involved in the attachment of the VTEC prototype strain NCTC 12900 to two gastrointestinal cell lines (HT29 and Caco-2) and was not identified in the attachment of the commensal *E. coli* strain HS. We also identified proteins, including AhpC and Enolase, which had been previously identified as immunogenic and/or involved in host cell attachment of VTEC, and/or other bacteria including *Burkholderia cenocepacia* and *Borrelia burgdorferi* [45,47,49], supporting the identification of the GlnH protein. Interestingly, Enolase, also referred to as Phosphopyruvate hydratase (Pph), a glycolytic enzyme, has previously been shown to employ moonlighting capabilities and plays a critical role in a variety of human diseases [50,51]. Moonlighting proteins are proteins that were initially considered to be solely conserved to specific cellular functions and have since been proven to have additional biological activities in locations in which they would not normally reside [52]. Enolase was identified as immunogenic in other invasive microorganisms, including *S. pneumoniae* [53,54] and *Campylobacter concisus* [55], and it contributes to the pathogenesis of *Borrelia burgdorferi* [49,56], the causative agent of Lyme disease. Therefore, it is possible that many of the proteins identified in our cell blots also possess moonlighting capabilities that are not yet fully understood and may include promising vaccine or therapeutic targets against VTEC serotypes and potentially other bacteria.

GlnH is a component of the glutamine transport system (GlnHPQ) and was initially considered to be localised to the periplasmic space [57]. It belongs to a larger superfamily of ATP-binding cassette (ABC) transporters [58], specifically from the bacterial extracellular solute-binding protein (SBP) cluster 3 [59], and is responsible for the active transport of _L_-glutamine across the cytoplasmic membrane [60]. Although predicted to be periplasmic, previous studies have demonstrated that GlnH possesses multifunctional capabilities in other locations. Bai et al. reported that GlnH was located in the extracellular milieu of *Salmonella* via outer membrane vesicles (OMVs) and confirmed its role in bacterial virulence and macrophage survival [61]. Similarly, a GlnH homologue, bacterial solute-binding protein 3, PEB1A, was characterised as a major cell adherence molecule and antigen of *Campylobacter jejuni* and as essential for the adhesion of *C. jejuni* to HeLa cells [62]. It was partially protective against *C. jejuni* as a vaccine in mice [63] and in chickens as a salmonella-vectored vaccine [64]. More recently, GlnH has been shown to be located in the proteo-surfaceome, the exoproteome and within extracellular vesicles of *E. coli* O157:H7 cells [65]. We confirmed that the GlnH protein has a role in *E. coli* attachment to human gastrointestinal epithelial cells, which supports the hypothesis that GlnH is a moonlighting protein involved in bacterial attachment to host cells. 

The immunisation of mice with purified rGlnH/Alum showed that it was a highly immunogenic antigen, as high IgG antibody titres were produced at both doses (10 µg and 50 µg), and the IgG2a/IgG1 ratio suggested a Th2-predominant response (Lin, L. et al., 2013). However, previous studies have shown that serological responses are not good predictors of protection against VTEC infection. Dziva et al. showed that strong antigen-specific IgG1 responses failed to have an impact on the magnitude or duration of the shedding of *E. coli* O157:H7 from calves [66]. Similarly, different combinations of antigenic vaccine formulations producing high antibody titres correlated with only partial protection against *E. coli* O157:H7 in cattle [67]. Indeed, our data suggest that a lower antigen dose eliciting a more moderate antibody response, together with a Th1 stimulating adjuvant, may be more effective in protecting against VTEC colonisation. The mixed Th1/Th2 response stimulated by GlnH/SAS correlated with a log 1.5-fold reduction in NCTC12900Nal^r^ CFU in the GI tracts of mice 7 d.p.i (*p* = 0.0280). This level of protection following immunisation with a single antigen is comparable to that achieved in mice immunised with DNA vaccine encoding 17 antigen candidates [28]. Overall, GlnH in combination with SAS reduced the level of O157 colonisation by 1.5 log relative to that of the GI tracts of mice treated with adjuvant only. This level of O157 reduction in the GI tract of mice confirmed that recombinant GlnH would be a suitable component in a multicomponent vaccine against VTEC. 

The NCTC12900 strain is a Vtx-negative strain that has previously been shown to colonise lambs and goats [37,68]. LaRegione et al. showed that NCTC 12900 Nal^r^ colonised the colon, caecum and rectum in 8-week-old goats and continued to be shed in faeces for up to 3 weeks [37]. Mahajan et al. subsequently showed that this strain adhered to bovine rectal epithelial cells [38], and we now show its attachment to two independent human gastrointestinal cell lines. Oral gavage of mice with NCTC 12900 Nal^r^ showed that faecal shedding and gastrointestinal colonisation were maintained in mice for up to 7 days. This oral challenge model with the monitoring of faecal shedding and gastrointestinal colonisation represents a safe and useful VTEC colonisation model in the absence of BLS3 animal facilities. Murine VTEC colonisation models are cost-effective models that are feasible for large sample sizes and have been used previously for VTEC vaccine testing [29,69,70], but they have limitations, as they are not naturally colonised by VTEC strains and thus do not develop symptoms [16,32]. Consequently, persistent levels of colonisation are not maintained after 7 days. Although human participants would offer an ideal model as the ultimate target population, this would not be feasible due to safety concerns. Ruminants could also be used as a model, but their use is costly and requires specialised facilities, which makes them less attractive for initial studies [16].

The correlates of protection and criteria for optimal protective responses against VTEC are poorly understood. Though previously identified protective vaccines induce a Th2-skewed response, it is recognised that cellular responses will also contribute to protection [16]. The impact of adjuvants on the protective responses cannot be underestimated. Moreover, although oral vaccination with subunit antigens showed suboptimal immunogenicity [71], a combination of systemic and mucosal immunisation may be optimal to protect against a gastrointestinal pathogen such as VTEC. Future studies will evaluate additional candidate vaccine antigens that were identified using our approach, both alone and in combination with GlnH. 

## 5. Conclusions

Overall, we have identified several adhesins involved in the attachment of VTEC strains to human gastrointestinal epithelial cells, and we have specifically located a vaccine candidate that offers protection against VTEC colonisation in mice. This highlights the effectiveness of our cell blot proteomic approach in identifying effective subunit protein antigens. 

## 6. Patents

This work has resulted in a patent application filed with the UK patent office. 

## Figures and Tables

**Table 1:** Proteins involved in host cell attachment of O157:H7 VTEC prototype strain and the commensal *E. coli* strain HS to two gastrointestinal cell lines from cell blots probed with HT29 or Caco-2.

No	Protein ID ^1^	Protein Name	Sequence Coverage (%)	Unique Peptides ^2^	pI ^3^	MW kDa ^4^	Score ^5^	Cell Line
1	P0AEQ5	Glutamine-binding periplasmic protein (GlnH)	77.8	21	8.44	27.19	358.58	HT29 & Caco-2
2	P65765	FKBP-type peptidyl-prolyl cis-trans isomerase FkpA	48.5	15	8.39	28.9	260.58	HT29 & Caco-2
3	P0AE10	Alkyl hydroperoxide reductase C (AhpC)	72.1	12	5.03	20.8	214.97	HT29 only
4	P0A6Q1	Enolase (Eno)	41	22	5.32	45.7	323.31	Caco-2 only
5	Q8XBL3	Phosphoenolpyruvate-protein phosphotransferase (PPP)	23.1	15	4.78	63.7	323.31	Caco-2 only
6	P0AGF1	Succinate—CoA ligase (ADP-forming) subunit alpha (SucD)	66.4	14	6.32	30.1	247.46	HT29 only
7	Q7ABI1	Protein GrpE	67.5	17	4.68	21.8	258.66	HT29 & Caco-2
8	Q8XD03	Phosphoglycerate kinase (PgK)	33.1	15	4.97	41.13	323.21	Caco-2 only
9	Q8XE41	Outer membrane protein C	14.4	10	4.6	40.51	260.11	HT29 & Caco-2
9a	P0A911	Outer membrane protein A	7.2	9	5.9	39.31	221.29	HT29 & Caco-2
10	Q8XAW6	D-Ribose periplasmic binding protein (RbsB)	67.9	16	6.85	30.9	304.08	HT29 only
11	Q8X8L3	Uridine phosphorylase (Udp)	83.3	12	5.71	27.3	218.33	HT29 only

^1^ Unitprot code; ^2^ number of unique peptides identified per sample; ^3^ theoretical isoelectric point; ^4^ molecular mass as determined by Q-Exactive LC-MS using the *E. coli* O157:H7 NCTC12900 database; ^5^ Spectrum Mill score at 1% FDR.

## Data Availability

The data presented in this study are available on request from the corresponding author. The data are not publicly available due to intellectual property concerns.

## Supplementary Materials

The following supporting information can be downloaded at: https://www.mdpi.com/article/10.3390/vaccines11010175/s1, Figure S1: Optimization of the NCTC12900 colonisation model.
