# Correction: Quinn et al. GlnH, a Novel Antigen That Offers Partial Protection against Verocytotoxigenic *Escherichia coli* Infection. *Vaccines* 2023, *11*, 175

**DOI:** 10.3390/vaccines11091414

**Published:** 2023-08-24

**Authors:** Conor Quinn, Julen Tomás-Cortázar, Oritsejolomi Ofioritse, Joanne Cosgrave, Claire Purcell, Catherine McAloon, Susanna Frost, Siobhán McClean

**Affiliations:** 1 School of Biomolecular and Biomedical Sciences, University College Dublin, Belfield, Dublin 4, Ireland; conor.quinn2@ucdconnect.ie (C.Q.); julen.tomascortazar@ucd.ie (J.T.-C.);; 2 UCD Conway Institute, University College Dublin, Belfield, Dublin 24, Ireland; 3 APC Ltd., Building 11, Cherrywood Business Park, Loughlinstown, D18 DH5 Co. Dublin, Ireland; 4 Children’s Health Ireland (CHI) at Tallaght, Tallaght University Hospital, Tallaght, Dublin 24, Irelandsusanna.frost@tuh.ie (S.F.); 5 School of Veterinary Medicine, University College Dublin, Belfield, Dublin 4, Ireland; catherine.mcaloon@ucd.ie

## Text Correction

In the original publication [1] there were two errors in text descriptions of the data that were presented in Figure 5B, but not in the data presented. 

The mean values for bacterial colonisation following either SAS and SAS + GlnH immunisation are log_10_ 4.45 and log_10_ 3.19 CFU per gram of colon, respectively, which are clearly apparent in the grey bars in Figure 5B and have not changed. However, this amounts to a 1.25-log reduction in the antigen immunised (SAS + GlnH) group, but was reported incorrectly as a 1.5-log reduction (which overstates the protection potential slightly), in the Abstract, Section 3.6 (Paragraph 2), and again in Section 4 (Paragraph 3). The statistical significance is maintained, and the *p* value is also unchanged.

In addition, this reduction in bacterial CFU was also inadvertently referred to as a 1.5-fold reduction in Section 3.6 (Paragraph 2) and Section 4 (Paragraph 3), which is incorrect. The antilog of log_10_ 4.45 = 28,183 and while the antilog of log_10_ 3.193 = 1560. This is clearly an 18-fold reduction in bacterial colonisation between the SAS treated group to the SAS + GlnH immunised group. 

Corrections have been made to the Abstract, Section 3.6 (Paragraph 2), and Section 4 (Paragraph 3):

1. In Abstract, the text: “with a 1.5-log reduction in colonisation of the colon and caecum at 7 days relative to the adjuvant only (*p* = 0.0280)” should be replaced with “with a 1.25-log reduction in colonisation of the colon and caecum at 7 days relative to the adjuvant only (*p* = 0.0280)”.
2. In Section 3.6, Paragraph 2, the text: “A 1.5-fold reduction in gastrointestinal colonisation (caecum and colon tissues combined, *p* = 0.0280)” should be replaced with “A 18-fold reduction in gastrointestinal colonisation (caecum and colon tissues combined, *p* = 0.0280)”.
3. In Section 4, Paragraph 3, the text: “The mixed Th1/Th2 response stimulated by GlnH/SAS correlated with a log 1.5-fold reduction in NCTC12900Nal^r^ CFU in the GI tracts of mice 7 d.p.i (*p* = 0.0280)” should be replaced with “The mixed Th1/Th2 response stimulated by GlnH/SAS correlated with a log 18-fold reduction in NCTC12900Nal^r^ CFU in the GI tracts of mice 7 d.p.i (*p* = 0.0280)”.
  “Overall, GlnH in combination with SAS reduced the level of O157 colonisation by 1.5 log relative to that of the GI tracts of mice treated with adjuvant only” should be replaced with “Overall, GlnH in combination with SAS reduced the level of O157 colonisation by 1.25 log relative to that of the GI tracts of mice treated with adjuvant only”.

The authors state that the scientific conclusions are unaffected. This correction was approved by the Academic Editor. The original publication has also been updated.
