# Supplementary figures and images for: GlnH, a Novel Antigen That Offers Partial Protection against Verocytotoxigenic Escherichia coli Infection
Source: Vaccines (Basel). 2023 Jan 13;11(1):175. doi: 10.3390/vaccines11010175 (PMC9863631; doi:10.3390/vaccines11010175)

# Optimisation of the NCTC12900 NaI<sup>r</sup> colonisation model.

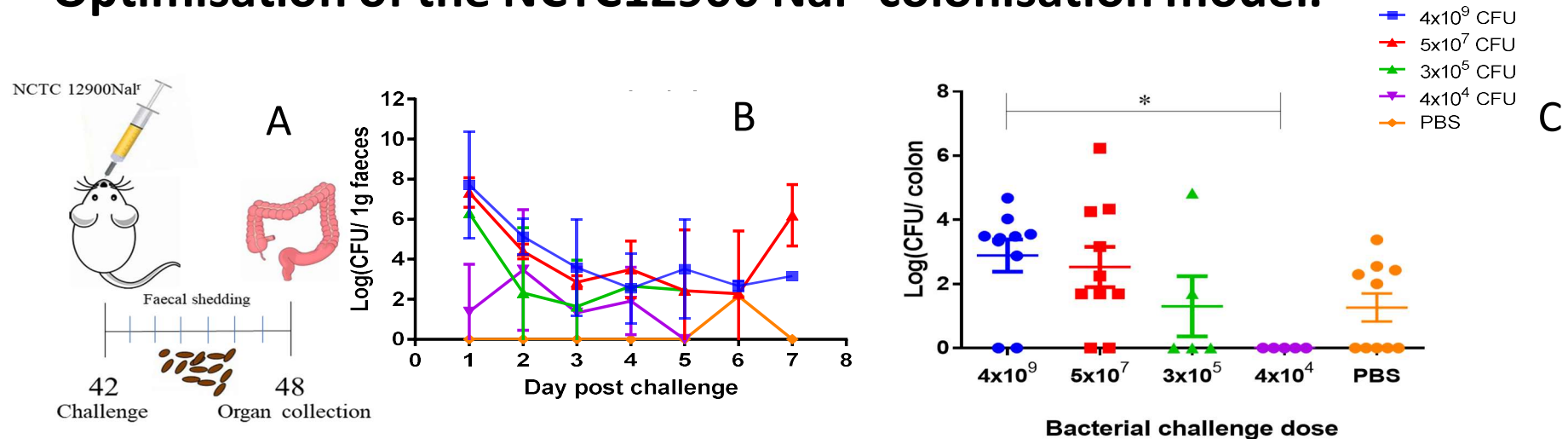

Supplement: Supplementary file 1 [file vaccines-11-00175-s001.zip › vaccines-2076653-supplementary.pdf]
